# Gut Dysbiosis as a Potential Guide for Immunotherapy (Dis)Continuation After 2 Years in NSCLC: A Brief Report

**DOI:** 10.1016/j.jtocrr.2025.100928

**Published:** 2025-10-29

**Authors:** Adele Bonato, Claudia Parisi, Priscilla Cascetta, Anna Reni, May-Lucie Meyer, Mariona Riudavets, David Planchard, Benjamin Besse, Jordi Remon, Francesco Facchinetti, Lorenzo Belluomini, Lisa Derosa, Fabrice Barlesi

**Affiliations:** a Department of Cancer Medicine, Gustave Roussy Cancer Medicine, Villejuif, France; b Department of Cancer Medicine, Paris-Saclay University, Ile-de-France, France; c ClinicObiome, Institut National De La Santé Et De La Recherche Médicale (INSERM) U1015, Gustave Roussy Cancer Campus, Villejuif, France; d Medical Oncology Department, Azienda Ospedaliero Universitaria (AOU) Pisana–Stabilimento di Santa Chiara, Pisa, Italy; e Department of Medical and Surgical Sciences and Translational Medicine, St. Andrea University Hospital, Sapienza University, Rome, Italy; f Medical Oncology Division, Cliniche Humanitas Gavazzeni, Bergamo, Italy; g Section of Innovation Biomedicine–Oncology Area, Department of Engineering for Innovation Medicine (DIMI), University of Verona, Italy; h Department of Oncology, Centre Hospitalier Universitarie Vaudois, Lausanne University, Lausanne, Switzerland

**Keywords:** NSCLC, Immunotherapy, Duration, (Dis)continuation, Gut microbiome

## Abstract

**Background:**

Although most phase III pivotal trials have set the duration of immune checkpoint blockers (ICB) for advanced NSCLC at 2 years, the criteria for safely discontinuing ICB remain undefined. Growing evidence links ICB efficacy to gut microbiota, positioning gut microbial taxonomic profiling as a promising biomarker to guide treatment decisions. We performed a retrospective analysis exploring clinical outcomes and the utility of multiomic decision-making tools in patients with NSCLC at Gustave Roussy who completed 24 months of ICB-based therapy without disease progression (PD).

**Methods:**

Patients receiving ICB between July 2016 and January 2023 were identified from the ONCOBIOTICS (NCT04567446) and STING (NCT04932525) study datasets. We selected those who reached 24 months of treatment without disease progression. Clinical characteristics and multiomic assessments, including gut microbiota profiling (TOPOSCORE by whole-genome sequencing), positron emission tomography–18-fluorodeoxyglucose imaging, and circulating tumor DNA, collected at 24 months, were analyzed. Key outcomes included overall survival (OS), progression-free survival (PFS), and PFS rates at 24 months after the completion of 2 years of ICB, stratified by molecular, metabolic, and microbial signatures.

**Results:**

Out of 123 patients treated for at least 18 months, 35 completed 24 months, with 31 eligible for the analysis. Of these, 68% continued ICB, whereas 32% discontinued therapy at the physician’s decision. Clinical characteristics were similar across groups. After a median follow-up of 59.1 months, OS and PFS did not differ significantly between those who discontinued and those who continued treatment (OS *p* = 0.9012). Among all multiomic tools, gut microbiota composition exhibited a trend (though not statistically significant) association with PFS rates at 24 months after the completion of 2 years of ICB. Patients with a favorable microbiota profile had a higher rate of sustained response at 24 months compared with those with dysbiotic signatures (81% versus 44%, respectively, *p* = 0.0870).

**Conclusions:**

Discontinuing ICB after 24 months did not negatively impact OS in our real-world cohort. Although limited by the small sample size, these findings support the potential of gut microbiota profiling as a promising tool to guide ICB duration. Integrating a translational multiomic algorithm, in particular microbial signals, may help personalize treatment strategies and safely shorten immunotherapy courses.

## Introduction

The recommended duration of Immune Checkpoint Blockers (ICB) as first-line treatment for NSCLC is 2 years, according to current clinical guidelines.^1^ Yet, even a decade after the first ICB approval for solid tumors, clinicians are reluctant to stop an active therapy after 2 years, and the debate on fixed-duration versus continuous treatment is still unsolved. The decision is often balanced between the patient’s fear of recurrence against concerns over cost and the uncertain risk of long-term toxicities. Although extended follow-up from KEYNOTE-189 and KEYNOTE-407 reported a 5-year overall survival (OS) rate of approximately 70% for patients completing 2 years of therapy,^2^^,^^3^ nearly half experienced disease progression after discontinuation.^3^^,^^4^ A recent retrospective study by Sun et al.^5^ on 706 patients with NSCLC treated with frontline immunotherapy found no survival benefit in continuing or stopping ICB at 2 years.

With the lack of reliable predictive biomarkers or clear discontinuation guidelines, clinicians rely on clinical and biological features such as programmed death-ligand 1 (PD-L1) expression, smoking status, tumor histology, and baseline tumor burden for (de)escalation proposal. Notably, smoking status correlates with survival in patients with NSCLC treated with ICB monotherapy, with active or former smokers achieving better outcomes than never-smokers, regardless of PD-L1 expression.^6^

Beyond clinical characteristics, additional tools could help decision-making on (dis)continuing ICB in NSCLC. Circulating tumor DNA (ctDNA) dynamics, for example, can anticipate disease progression and response to immunotherapy weeks to months before conventional imaging.^7^ Ricciuti et al.^7^ identified ctDNA allele fraction (AF) as an early pharmacodynamic biomarker of response or resistance to ICB, in which a drop in AF between pretreatment and first on-treatment blood draw was linked to higher response rates, longer progression-free survival (PFS), and OS.

Functional imaging by means of positron emission tomography–18-fluorodeoxyglucose (FDG-PET) may help identify patients at higher risk of relapse after ICB discontinuation, such as those who fail to reach complete metabolic response (CMR).^8^ This was largely exhibited in metastatic melanoma, but data on NSCLC are limited. The multicentric retrospective INTEPI study evaluated Patients with NSCLC who discontinued ICB monotherapy after 18 months with disease control: PFS rates with complete response at computed tomography scan or CMR at FDG-PET postdiscontinuation were 71% and 66% at 12 and 24 months, respectively.^9^

The gut microbiome has also emerged as a key determinant of immunotherapy efficacy, making microbial profiling a promising predictive biomarker for ICB response.^10^ Interestingly, to account for the bacterial ecosystem variability, Derosa et al.^10^ developed the TOPOSCORE, a metric combining the ratio of two species-interacting groups (SIGs, SIG1, and SIG2, calculated before starting immunotherapy, and associated with poor and favorable survival, respectively) with the relative abundance of *Akkermansia muciniphila,* a bacterium previously linked to prognosis in patients with NSCLC receiving ICB.

Given the lack of definitive data on optimal immunotherapy duration, we conducted a retrospective study evaluating clinical outcomes and decision-making tools used by thoracic oncologists at Gustave Roussy for patients with NSCLC who completed 24 months of ICB-based treatment without progressive disease (PD). We retrieved blood and stool samples collected at 24 months for multiomic analyses, including ctDNA, and gut microbiome profiling. In this context, the TOPOSCORE was applied to the stool samples collected at the 24-month time point to stratify patients according to gut microbiota composition.

## Materials and Methods

Patients diagnosed with advanced NSCLC and treated with ICB between July 2016 and January 2023 were identified from ONCOBIOTICS (NCT04567446) and STING trials (NCT04932525). We selected those who had reached at least 24 months (range 23.5–29.7) of treatment in the absence of PD. For these patients, stool and blood samples were prospectively collected as part of the study protocol. We assessed clinical characteristics using descriptive statistics. The OS and PFS curves were estimated using the Kaplan-Meier method and compared with the log-rank test. The PFS rate at 24 months after the completion of 2 years of ICB (PFS24) was also assessed and stratified on the basis of multiomic analyses, including gut-based biomarkers (i.e., TOPOSCORE by whole-genome sequencing), FDG-PET imaging, and ctDNA. We classified patients who achieved PFS24 as “responders” and those who did not as “non responders.” CtDNA analysis was performed to detect circulating tumor DNA using both polymerase chain reaction and next-generation sequencing–based approaches. TOPOSCORE was computed using the same metagenomic sequencing, bioinformatic, and statistical analysis as in Derosa et al.^10^ Categorical variables were compared with two-tailed chi-square or Fisher’s exact tests. Statistical analyses were conducted using GraphPad Prism 10 Software (GraphPad, Boston, MA).

## Results

**Figure 1:**
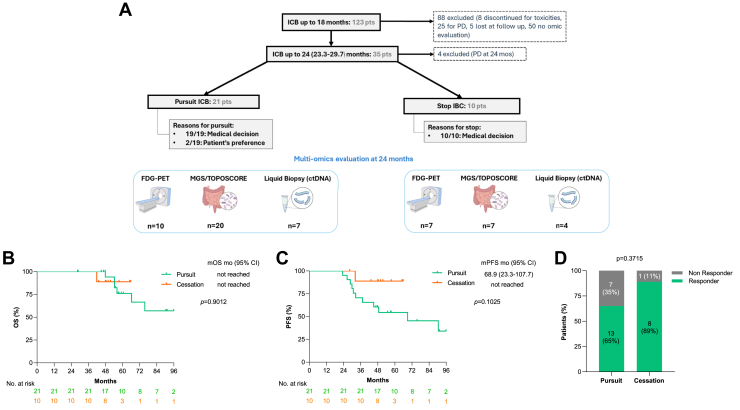
Patients’ enrollment and clinical outcomes on the basis of ICB duration and TOPOSCORE categories. (*A*) Flowchart illustrating patients’ inclusion, rational for treatment continuation or discontinuation and number of patients evaluated with different omics (FDG-PET, MGS, and ctDNA). (*B,C*) Cox regression univariable analysis and Kaplan-Meier survival curves for OS and PFS (*C*) between patients who continued (pursuit) and stopped (cessation) ICB at 24 months; (D**)** Bar graphs displaying the rates of PFS24 after 2 years of ICB between the pursuit and cessation groups, with “responder” identifying those who reached PFS24 and “non responder” those who did not. The *p* values were obtained with two-tailed chi-square or Fisher’s exact tests. ICB, immune checkpoint blockers; pts, patients; PD, progressive disease; FDG-PET, fluoro-d-glucose positron emission tomography; MGS, metagenomic sequencing; ctDNA, circulating tumor DNA; n, number; CI, confidence interval; PFS24, progression-free survival at 24 months from 24-months landmark.

In our cohort, 123 consecutive patients with advanced NSCLC received ICB for at least 18 months, 35 reached the 24-month landmark (Fig. 1*A*). Of these, 31 patients were included in the analysis after excluding four who experienced PD exactly at 24 months. Among the analyzed patients, 21 (68%) continued ICB beyond 2 years, whereas 10 (32%) stopped treatment between 23.5 and 29.7 months on the basis of the physician’s discretion. Multiomic data, including PET-FDG imaging, ctDNA, and stool metagenomics profiling stratified by TOPOSCORE,^10^ were collected at 24 months when available (Fig. 1*A*).

Baseline clinical characteristics collected before ICB start (i.e., sex, Eastern Cooperative Oncology Group Performance Status, tumor histology, line of treatment, or treatment regimen) were comparable between the two groups and remained consistent over time (Table 1). After a median follow-up of 59.1 months (range: 28.4–107.7), the median duration of treatment was 24.9 months (range: 23.5–29.7) for patients who stopped and 44.4 months (28.8–107.4) for those who continued. In accordance with the findings from other retrospective cohorts,^5^ we did not observe significant differences in OS (*p = 0.9012*) nor PFS (*p = 0.1025*) between the two groups (Fig. 1*B-C*).

**Table 1:** Clinical Characteristics of the Gustave Roussy Retrospective Cohort (N = 31)

Characteristics	All Patients	Cessation Group	Pursuit Group	*p*-Value^a^
	N = 31	N = 10	N = 21	
Gender - no. (%)				
Male	17 (55)	5 (50)	12 (57)	0.7366
Female	14 (45)	5 (50)	9 (43)	
Age years - median (range)	61 (39–77)	61 (39–68)	62 (43–77)	
ECOG performance status - no. (%)				
0	10 (32)	3 (30)	7 (33)	
1	14 (45)	6 (60)	8 (38)	0.2044
2	7 (23)	1 (10)	6 (29)	
Smoking status - no. (%)				
Never	0	0	0	
Former	24 (80)	9 (90)	15 (75)	>0.9999
Current	6 (20)	1 (10)	5 (25)	
Unknown	1	-	1	
Histology - no. (%)				
Adenocarcinoma	23 (74)	8 (80)	15 (71)	0.5893
Squamous	3 (10)	1 (10)	2 (10)	
Undifferentiated/Other	5 (16)	1 (10)	4 (19)	
PD-L1 expression - no. (%)				
<1%	2 (8)	0	2 (13)	0.5749
≥1%–<50%	5 (20)	3 (30)	2 (13)	
≥50%	18 (72)	7 (70)	11 (73)	
Unknown	6	-	6	
Treatment regimen - no. (%)				
Chemoimmunotherapy	5 (16)	3 (30)	2 (10)	0.1907
Monoimmunotherapy	26 (84)	7 (70)	19 (90)	
Line of treatment - no. (%)				
First	17 (55)	7 (70)	10 (48)	0.7332
≥ Second	14 (45)	3 (30)	11 (52)	
No. of metastatic sites - no. (%)				
1–2	18 (58)	6 (60)	12 (57)	0.9461
3–4	9 (29)	3 (30)	6 (29)	
>4	4 (13)	1 (10)	3 (14)	
Immune Related AE (any grade) no. (%)				
Yes	8 (31)	1 (13)	7 (47)	0.1784
No	18 (69)	7 (87)	8 (53)	
NA	5	2	6	

a Chi-Square test between cessation and pursuit groups.

**Figure 2:**
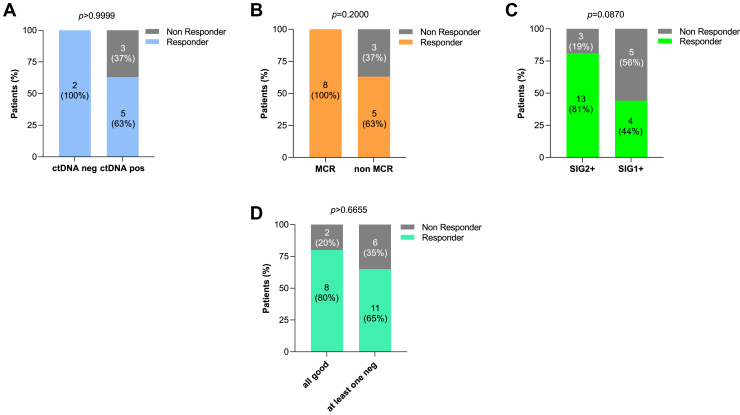
PFS24 rates on the basis of omics outcomes. (*A*) Bar graphs displaying the rates of PFS at 24 months from 24-months landmark (PFS24) between ctDNA-positive and ctDNA-negative groups. (*B*) Bar graphs illustrating PFS24 rates between patients that reached a MCR and those who did not (non-MCR). (*C*) Bar graphs illustrating the rates of PFS24 between favorable (SIG2+) and unfavorable microbiota score (SIG1+). (*D*) Same graph between patients whose available omics (FDG-PET and or ctDNA and or TOPOSCORE) all had a favorable outcome and patients with at least one omic result suggesting an unfavorable outcome. « Responder » identifies patients who reached PFS24 and « Non Responder » those who did not reach PFS24. The p-values were obtained with two-tailed Chi-square or Fisher’s exact tests. PFS24, progression-free survival at 24 months from 24-months landmark; MCR, metabolic complete response; FDG-PET, positron emission tomography–18-fluorodeoxyglucose; ctDNA, circulating tumor DNA.

As the median OS was not reached in both groups, we focused subsequent analyses on PFS from the 24-month landmark (PFS24). Again, no significant difference in PFS24 was observed between patients who stopped treatment and those who continued treatment beyond 2 years (*p = 0.3715*, Fig. 1*D*). To further dissect potential predictive tools, we investigated each multiomic assessment independently (Fig. 2). Whereas ctDNA (Fig. 2*A*) and PET-FDG (Fig. 2*B*) revealed no significant correlation with PFS24 (*p = 0.9999* and *p = 0.2000*, respectively), gut microbiota profiling (Fig. 2*C*), stratified by the TOPOSCORE-defined SIGs, suggested a trend toward predictive value. Specifically, patients with a favorable gut composition (SIG2+) exhibited a trend toward superior PFS24 rates compared with those with dysbiotic profile (SIG1+), with long responders comprising 81% and 44% of each group, respectively (*p* = 0.0870) (Fig. 2*C*). Notably, combining all multiomic tools did not enhance predictive performance beyond that of gut microbiota alone (Fig. 2*D*).

## Discussion

Physicians currently lack evidence-based tools to guide treatment decisions after 2 years of ICB for advanced NSCLC. Extended ICB use may lead to unnecessary social, safety, and economic strain. Retrospective data suggest no OS benefit from continuing ICB beyond 24 months.^5^ Supporting this, a recent French study involving 43,359 patients found no difference in mortality risk between those who continued or stopped pembrolizumab at 2 years.^11^ However, oncologists remain cautious, as other trials such as CheckMate 153 indicated worse PFS with a 1-year fixed treatment duration compared with continued treatment, even though progression may still occur regardless of treatment continuation.^12^

Several factors, including smoking status, ICB responses, immune-related adverse events rate, and genomic abnormalities (such as KRAS mutations), may help guide ICB cessation, though no consensus exists. CtDNA, which has a role in early-stage NSCLC, could be key to detecting residual disease post-ICB, and several studies like BESPOKE IO (NCT04761783) are exploring its predictive role and impact on clinical decision-making. FDG-PET also exhibited a potential predictive value, with CMR able to predict long-term benefit mainly in metastatic melanoma, in which a proposed ICB cessation algorithm integrates metabolic imaging and pathology of active lesions.^8^ Of note, a shift of gut microbiota taxonomic composition toward dysbiosis can be detrimental for clinical outcome during ICB in solid cancers.^13^^,^^14^ Particularly, TOPOSCORE, a unidimensional dysbiosis score, allows survival risk stratification of patients receiving ICB on the basis of known prognostic factors (such as PD-L1 and Eastern Cooperative Oncology Group Performance Status) and can also predict immune-related adverse events.^15^ In this light, given its proven clinical utility, temporal stability, and the fact that stool sample collection is both noninvasive and easy to perform, gut microbiota assessment, particularly by means of the TOPOSCORE, emerges as a feasible tool for guiding ICB continuation or discontinuation. Beyond TOPOSCORE, other promising, and easily implementable surrogate biomarkers of dysbiosis, such as serum levels of MAdCAM-1, may also play a role in this setting.^16^

On the basis of these premises, our analysis attempted to explore potential biomarkers that could predict clinical outcomes and inform decisions regarding ICB (dis)continuation decision. Among the tools evaluated, gut microbiota scores detecting dysbiosis emerged as a promising approach to guide treatment cessation.

However, our study has several limitations. First, the small sample size limits the strength and generalizability of our conclusions. Second, more than 65% of patients in our cohort pursued treatment beyond 2 years, despite the lack of strong clinical evidence supporting this practice. This decision was often shaped by shared oncologist-patient discussion, in which individual patient preferences often played a central role. Third, our study population consisted exclusively of “considerable responders” patients, defined as patients who achieved 24 months of ICB treatment without disease progression. As a result, no internal comparator group of “non considerable” responders could be included by design. Additional limitations include its retrospective design and the lack of complete multiomic data for the entire cohort. To address these gaps, a larger prospective study is strongly needed to validate gut microbiota assessment by means of TOPOSCORE as a clinical tool to inform treatment duration, and even de-escalation, of ICB in optimally selected patients.

## CRediT Authorship Contribution Statement

**Adele Bonato:** Conceptualization, Methodology, Resource, Software, Data curation, Writing - original draft, Visualization, Writing - review & editing.

**Claudia Parisi:** Conceptualization, Methodology, Resource, Data curation, Writing - original draft, Visualization, Writing - review & editing.

**Priscilla Cascetta:** Resource, Data curation, Writing - original draft, Visualization, Writing - review & editing.

**Anna Reni:** Resource, Data curation, Writing - original draft, Visualization, Writing - review & editing.

**May-Lucie Meyer:** Resource, Data curation, Writing - original draft, Visualization, Writing - review & editing.

**Mariona Riudavets:** Resource, Data curation, Writing - original draft, Visualization, Writing - review & editing.

**David Planchard:** Resource, Data curation, Writing - original draft, Visualization, Writing - review & editing.

**Benjamin Besse:** Resource, Data curation, Writing - original draft, Visualization, Writing - review & editing.

**Jordi Remon:** Resource, Data curation, Writing - original draft, Visualization, Writing - review & editing.

**Francesco Facchinetti:** Resource, Data curation, Writing - original draft, Visualization, Writing - review & editing.

**Lorenzo Belluomini:** Conceptualization, Methodology, Resource, Software, Data curation, Writing - original draft, Visualization, Writing - review & editing.

**Lisa Derosa:** Conceptualization, Methodology, Resource, Software, Data curation, Writing - original draft, Visualization, Writing - review & editing.

**Fabrice Barlesi:** Conceptualization, Methodology, Resource, Software, Data curation, Writing - original draft, Visualization, Writing - review & editing.

## Disclosure

Dr. Belluomini received speakers’ fees from AstraZeneca, Merck Sharp & Dohme, Roche, Takeda, Novartis, and BMS outside the submitted work; received travel fees from Takeda outside of the submitted work; and is an EverImmune SAB member. Dr. Facchinetti received a speaker's fee from Roche and participated in an advisory board for BeiGene outside of the submitted work. The remaining authors declare no conflict of interest.

## References

[1] Reck M., Remon J., Hellmann M.D. (2022). First-line immunotherapy for non-small-cell lung cancer. J Clin Oncol.

[2] Novello S., Kowalski D.M., Luft A. (2023). Pembrolizumab plus chemotherapy in squamous non-small-cell lung cancer: 5-year update of the Phase III KEYNOTE-407 study. J Clin Oncol.

[3] Garassino M.C., Gadgeel S., Speranza G. (2023). Pembrolizumab plus pemetrexed and platinum in nonsquamous non-small-cell lung cancer: 5-year outcomes from the Phase 3 KEYNOTE-189 study. J Clin Oncol.

[4] Reck M., Rodríguez-Abreu D., Robinson A.G. (2021). Five-year outcomes with pembrolizumab versus chemotherapy for metastatic non-small-cell lung cancer with PD-L1 tumor proportion score ≥ 50. J Clin Oncol.

[5] Sun L., Bleiberg B., Hwang W.T. (2023). Association between duration of immunotherapy and overall survival in advanced non-small cell lung cancer. JAMA Oncol.

[6] Gainor J.F., Rizvi H., Jimenez Aguilar E. (2020). Clinical activity of programmed cell death 1 (PD-1) blockade in never, light, and heavy smokers with non-small-cell lung cancer and PD-L1 expression ≥50. Ann Oncol.

[7] Ricciuti B., Jones G., Severgnini M. (2021). Early plasma circulating tumor DNA (ctDNA) changes predict response to first-line pembrolizumab-based therapy in non-small cell lung cancer (NSCLC). J Immunother Cancer.

[8] Dimitriou F., Lo S.N., Tan A.C. (2022). FDG-PET to predict long-term outcome from anti-PD-1 therapy in metastatic melanoma. Ann Oncol.

[9] Bilger G., Girard N., Doubre H. (2022). Discontinuation of immune checkpoint inhibitor (ICI) above 18 months of treatment in real-life patients with advanced non-small cell lung cancer (NSCLC): INTEPI, a multicentric retrospective study. Cancer Immunol Immunother.

[10] Derosa L., Iebba V., Silva C.A.C. (2024). Custom scoring based on ecological topology of gut microbiota associated with cancer immunotherapy outcome. Cell.

[11] Rousseau A., Michiels S., Simon-Tillaux N. (2024). Impact of pembrolizumab treatment duration on overall survival and prognostic factors in advanced non-small cell lung cancer: a nationwide retrospective cohort study. Lancet Reg Health Eur.

[12] Waterhouse D.M., Garon E.B., Chandler J. (2020). Continuous versus 1-year fixed-duration nivolumab in previously treated advanced non-small-cell lung cancer: CheckMate 153. J Clin Oncol.

[13] Silva C.A.C., Fidelle M., Almonte A.A., Derosa L., Zitvogel L. (2025). Gut microbiota-related biomarkers in immuno-oncology. Annu Rev Pharmacol Toxicol.

[14] Derosa L., Routy B., Thomas A.M. (2022). Intestinal Akkermansia muciniphila predicts clinical response to PD-1 blockade in patients with advanced non-small-cell lung cancer. Nat Med.

[15] Vitali G., Silva C.A.C., Oudabi D. (2025). Association of intestinal exfoliome and Prevotellaceae with toxicity and clinical outcome during immune-checkpoint blockade. J Clin Oncol.

[16] Saliby R.M., Machaalani M., Zhong C. (2025). Gut-associated checkpoint as a prognostic biomarker in metastatic renal cell carcinoma (mRCC): results from a randomized first-line clinical trial. J Clin Oncol.

